# Research waste of randomized controlled trials related to deep brain stimulation: a cross-sectional analysis

**DOI:** 10.3389/fnagi.2026.1818434

**Published:** 2026-07-10

**Authors:** Qiu Zeng, Xinxing Fei, Qiuyu Zhou, Weiyi Li, Jianxiong Wang, Yaqian Gao, Yue Hu

**Affiliations:** 1 Department of Rehabilitation Medicine, The Sixth People’s Hospital of Yibin, Yibin, Sichuan, China; 2 Department of Psychiatry, Chengdu Eighth People’s Hospital (Geriatric Hospital of Chengdu Medical College), Chengdu, China; 3 Department of Rehabilitation Medicine, The Affiliated Hospital of Southwest Medical University, Luzhou, China; 4 Rehabilitation Medicine and Engineering Key Laboratory of Luzhou, Luzhou, China; 5 Department of Rehabilitation Medicine, The First Affiliated Hospital of Chengdu Medical College, Chengdu, China; 6 Dipartimento di Psicologia, Centro Studi e Ricerche in Neuroscienze Cognitive, Alma Mater Studiorum–Università di Bologna, Cesena, Italy

**Keywords:** cross-sectional analysis, deep brain stimulation, multicenter, randomized controlled trials, research waste

## Abstract

**Background:**

Research waste undermines the value of clinical trials. This study aimed to quantify its prevalence, characterize its forms, and identify associated factors in phase III/IV randomized controlled trials (RCTs) of deep brain stimulation (DBS).

**Methods:**

We conducted a cross-sectional analysis of DBS trials registered on ClinicalTrials.gov. Research waste was defined as non-publication, inadequate reporting, or avoidable design flaws. Trial characteristics were extracted from registry entries. Publication status was ascertained by searching the PubMed, Embase, and Scopus databases. The chi-square or Fisher’s exact test was used to compare groups, and logistic regression was used to identify factors associated with outcomes.

**Results:**

Among 26 eligible RCTs, the non-publication rate was 65.38%. Only 5 trials (19.23%) were free of research waste. All published trials and all waste-free trials were registered before 2009, whereas 64.71% of unpublished trials were registered in 2010 or later (*P* = 0.002). International recruitment was significantly associated with reduced waste (*P* = 0.004). Univariate analysis suggested multicenter trials (*P* = 0.027) and larger sample sizes (*P* = 0.015) were more likely to be published; however, these associations did not retain significance in multivariate models, likely due to limited statistical power.

**Conclusion:**

Research waste is a widespread phenomenon in DBS RCTs. Effective coordination and resource management are paramount in translating DBS research into publicly accessible evidence.

## Introduction

Deep brain stimulation (DBS) is a well-established and rapidly evolving neurosurgical intervention for a variety of neurological disorders, such as Parkinson’s disease, epilepsy, and dystonia, and psychiatric disorders, including obsessive-compulsive disorder and treatment-resistant depression (Dougherty, 2018; Neumann et al., 2023; Steigerwald et al., 2019). The clinical adoption and refinement of DBS protocols for these diverse indications are predominantly driven by evidence generated from large-scale randomized controlled trials (RCTs), particularly those in phases III and IV, which are designed to confirm efficacy and monitor long-term outcomes in broader patient populations (Chapman et al., 2019; Pandis et al., 2021).

Despite their pivotal role, the full value of these critical trials is often compromised by pervasive research waste. This encompasses the non-publication of completed study results, inadequate reporting that hinders interpretation and replication, and avoidable methodological flaws in trial design or conduct (Liu et al., 2014; Taljaard et al., 2011). Notably, while a certain level of non-publication may be anticipated or even accepted for early phase (phase I/II) trials that are primarily exploratory, such attrition is considered a more significant form of waste in later-phase (phase III/IV) trials, which are intended to provide definitive evidence to guide clinical practice (Chamberlain et al., 2025; Fei et al., 2026; Zhao et al., 2025). This susceptibility is particularly acute in neuromodulation research due to protracted recruitment needs, the logistical complexities of multi-center surgical standardization, and the frequent misalignment between lengthy trial cycles and rapid device innovation (Mishra et al., 2024). Such waste leads to the inefficient use of substantial financial resources, undermines the contributions and risks undertaken by research participants, and ultimately delays the translation of reliable evidence into clinical practice, posing significant scientific and ethical challenges for the field (Zheutlin et al., 2020).

Currently, a comprehensive assessment of the characteristics and extent of research waste in DBS-related phase III and IV RCTs is lacking. Quantifying this issue is essential to understanding the current state of evidence generation, identifying systemic weaknesses, and promoting strategies that maximize the return on research investment. Therefore, this cross-sectional analysis aims to systematically characterize the landscape of these trials, quantify the prevalence and forms of research waste, and identify factors associated with the production of robust, usable, and translatable clinical evidence.

## Materials and methods

### Study design and data source

This cross-sectional analysis followed the STROCSS guidelines for reporting (Rashid et al., 2024). The data were extracted from the public clinical trial registry ClinicalTrials.gov. A systematic database search was performed on June 20, 2026, using a predefined strategy that included the terms “Deep Brain Stimulations,” “Deep Brain Stimulation,” “Electrical Stimulation of the Brain,” and “DBS” entered into the Intervention/treatment field. The search was limited to Phase III and Phase IV trials using the platform’s built-in filter, with the study completion date set to December 31, 2024, accounting for the potential 1-year interval between research completion and publication. Following the export of the filtered search results to Excel, non-randomized trials or those unrelated to DBS were manually excluded during the screening process. Minor variations in the total number of screened records may occur due to ongoing updates and maintenance of the ClinicalTrials.gov registry interface.

### Data extraction and publications identification

For each eligible RCT, the following characteristics were recorded from the registry entry: ClinicalTrials.gov identifier (NCT number), study title, study start and completion dates, intervention details, condition, primary purpose, study design, allocation, masking information, number of arms, enrollment size, country/location of the principal investigator, participating centers, and funding source. To determine the publication status of each registered RCT, we implemented the following steps: First, we searched PubMed, Embase, and Scopus. The search strategy combined the trial’s NCT identifier, the principal investigators’ names, and keywords related to the intervention and condition. If no corresponding publication was found via the database search, we attempted to contact the corresponding or lead investigator to confirm its publication status. Finally, a trial was classified as “published” if a full manuscript was available in a peer-reviewed journal.

### Assessment of adequate reporting

Two investigators (QZ and XF) independently evaluated the quality of reporting for each published trial included in this study. Both reviewers are co-authors of the present manuscript but had no involvement in, or personal interest in, any of the DBS trials under assessment, thereby avoiding potential conflicts of interest. In case of any disagreement, they would resolve it through discussion. Given that DBS is primarily a device-based, non-pharmacological intervention, the assessment was based on the relevant items of the Consolidated Standards of Reporting Trials (CONSORT) 2010 statement, with particular attention to its extensions for non-pharmacological treatment trials (Piaggio et al., 2012). Although the more recent CONSORT 2025 guidelines exist, the CONSORT 2010 version was selected for this analysis because most of the eligible RCTs in our cohort were conducted and reported before the updated guidelines were released (Chan et al., 2025). Each checklist item was scored as either “reported” or “not reported.” Any discrepancies between the two reviewers were resolved through a consensus discussion. A trial publication was considered to have adequate reporting if it met at least 3/4 of the applicable items on the CONSORT checklist.

### Assessment of avoidable design flaws

Two investigators (QZ and XF) independently assessed the risk of bias for each published RCT using the Cochrane Risk of Bias tool (Higgins et al., 2011). The tool evaluates key domains, including the randomization process, deviations from intended interventions, handling of missing outcome data, measurement of the outcome, and selection of the reported result. Any trial that was rated as having an “unclear” or “high” risk of bias in one or more domains was categorized as having an avoidable design flaw.

### Definition of research waste

In line with the methodology of other research in this field, research waste was operationally defined for this study (Chen et al., 2024). An RCT was classified as exhibiting research waste if it met one or more of the following criteria: (1) non-publication of its results in a peer-reviewed journal; (2) upon publication, providing inadequate reporting that fell below the adherence threshold of at least three-fourths of the applicable CONSORT checklist items; or (3) for published trials, possessing an avoidable design flaw, as determined by an assessment yielding a judgment of “unclear risk” or “high risk” in at least one methodological domain evaluated by the Cochrane Risk of Bias tool. This composite definition aims to capture the spectrum of resource inefficiency, from trials whose results remain unseen to those whose reported evidence is compromised in quality.

### Statistical analysis

Descriptive statistics summarized the data. Group comparisons for categorical variables were performed using the chi-square or Fisher’s exact test, as appropriate. Univariate and multivariate logistic regression analyses were used to identify factors independently associated with research waste. Factors with *P* < 0.2 in univariate analysis were entered into the multivariate model. Forest plots and stacked bar charts were generated using R (Version 4.5.1). All analyses were conducted using GraphPad Prism (Version 10.1.2), with a two-sided *P* < 0.05 considered statistically significant.

## Results

### Study selection

**FIGURE 1:**
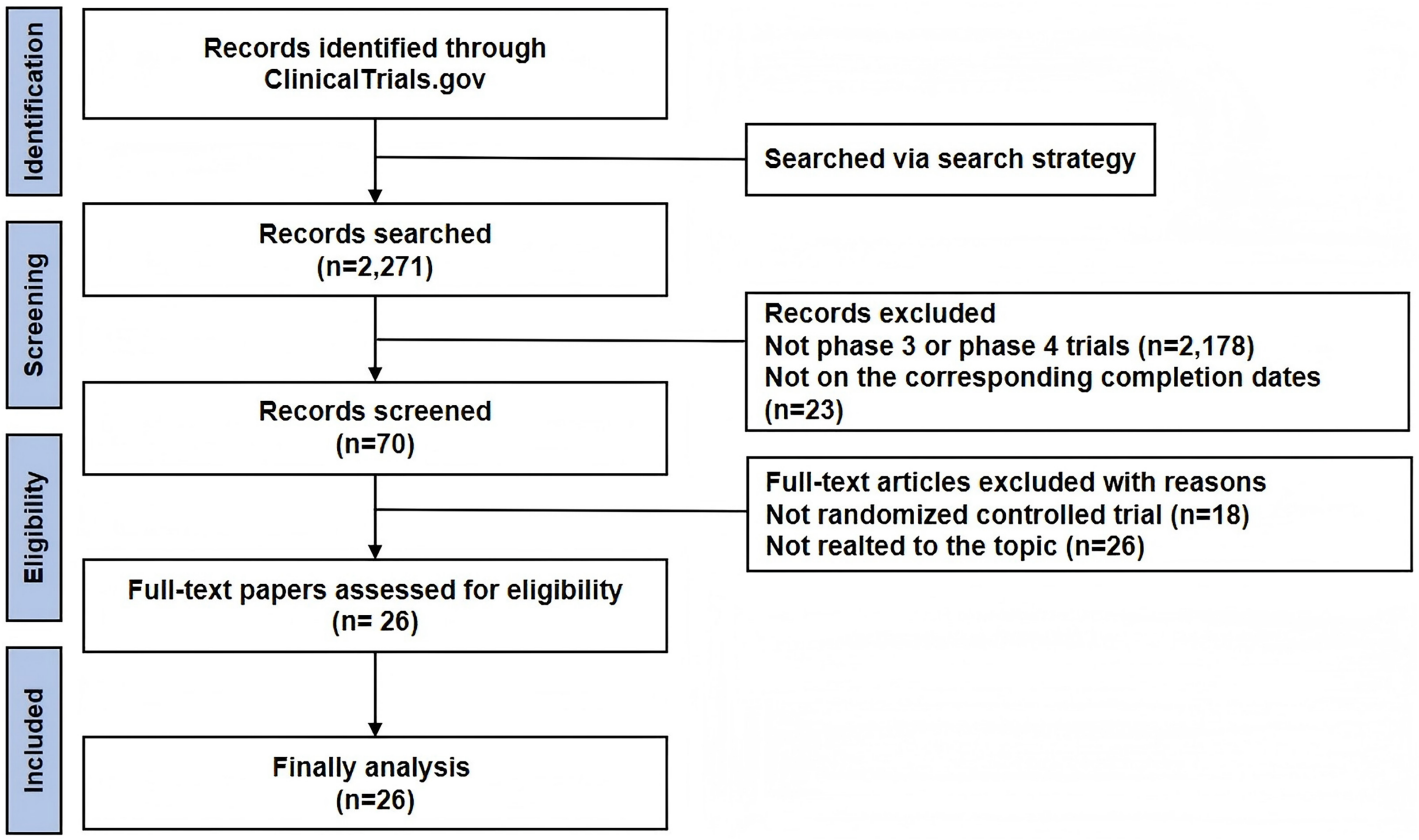
Flow chart of study identification.

The systematic search of the ClinicalTrials.gov database yielded an initial 2,271 records. Following the removal of trials that were not phase III or IV (*n* = 2,178) and those that did not meet the specified completion-date criteria (*n* = 23), 70 records were screened for eligibility. After a full-text review, 44 records were excluded for the following reasons: they were not RCTs (*n* = 18) or not relevant to DBS (*n* = 26). Ultimately, 26 unique RCTs met all inclusion criteria and were included in the final cross-sectional analysis. The study selection process is detailed in the PRISMA flow diagram (Figure 1).

### Characteristics of included RCTs

A total of 26 phase III and IV RCTs related to DBS were ultimately included in the analysis. The characteristics of these trials are summarized in Table 1. Overall, a substantial portion of the trials (57.69%) were registered before 2010, indicating a historical concentration of DBS efficacy trials in the late 2000s. The majority of trials focused on neurological disorders, with a smaller yet significant subset investigating psychiatric indications, reflecting the evolving application of DBS. The trial design was predominantly conventional, with parallel groups and a two-arm structure. Blinding practices varied widely, with double-blinding being the most common but not the universal standard. Notably, the included RCTs were characterized by a high degree of non-departmental sponsorship. The sample sizes of the trials were typically small; the median number of participants was 29, with an interquartile range (IQR) of 8–99. Based on this distribution, 20 trials (76.92%) enrolled fewer than 100 participants, while only 23.08% enrolled 100 or more. Most RCTs were single-center, nationally conducted studies. Europe was the primary region for these trials, followed by North America and Asia.

**TABLE 1:** Characteristics of RCTs related to DBS.

Characteristic	No. of RCTs (26)	Proportion
Start time		
Before 2009 (inclusive)	15	57.69%
After 2010 (inclusive)	11	42.31%
Condition		
Neurological disorders	19	73.08%
Psychiatric/psychological disorders	7	26.92%
Study design		
Parallel group	21	80.77%
Non-parallel group	8	30.77%
No. of arms		
2	24	92.31%
≥ 3	2	7.69%
Blinding		
None/open label	6	23.08%
Single	3	11.54%
Double	8	30.77%
Triple	3	11.54%
Quadruple	6	23.08%
Recruitment		
National	23	88.46%
International	3	11.54%
No. of centers		
Single center	14	53.85%
Multicenter	12	46.15%
No. of participants		
<100	20	76.92%
≥100	6	23.08%
Region of PI		
North America	7	26.92%
Europe	15	57.69%
Asia	4	15.38%
Funding		
None or departmental	2	7.69%
Industry or other external	24	92.31%

PI, principal investigator.

### Publication status of RCTs

**FIGURE 2:**
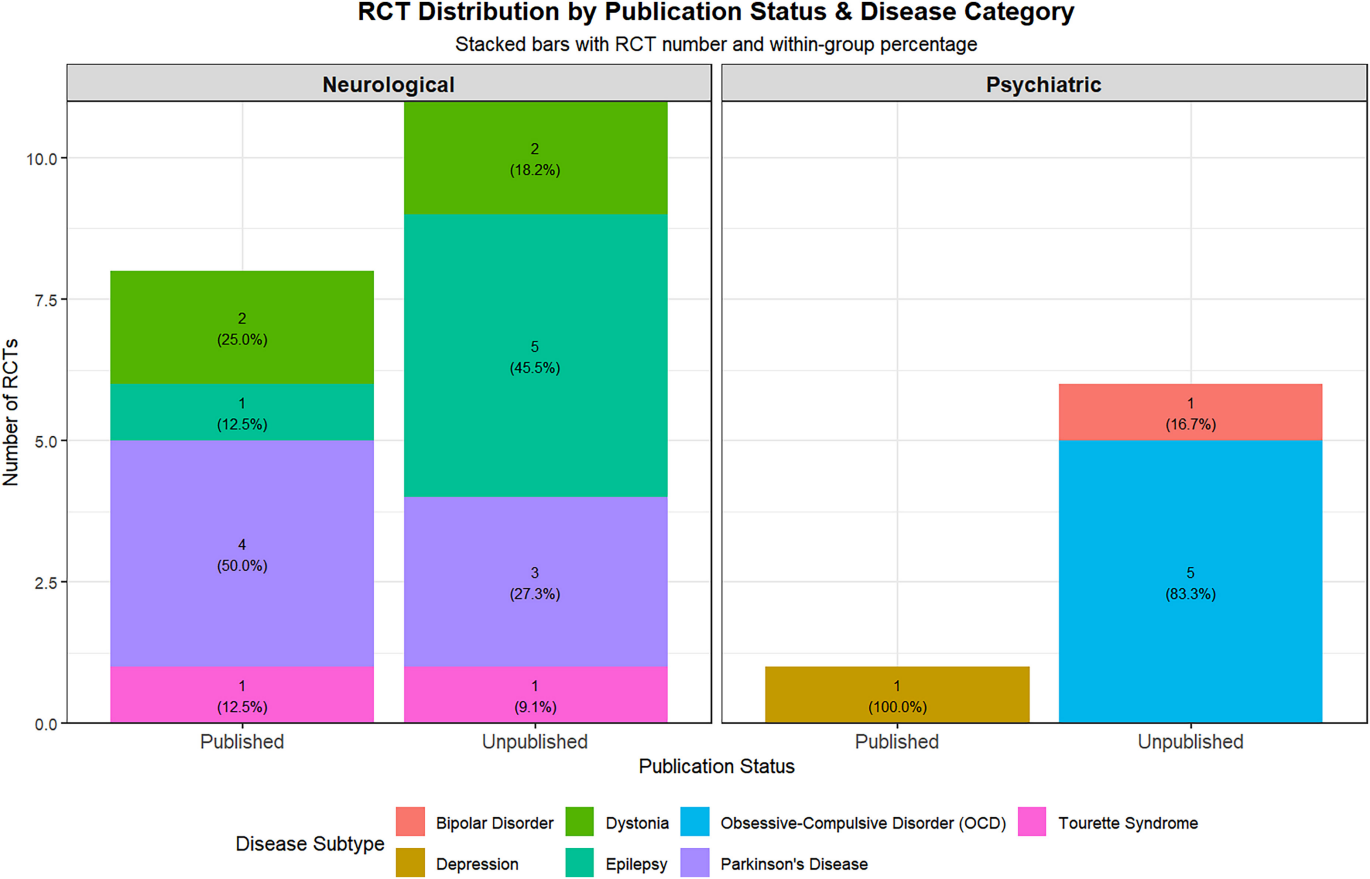
Distribution of registered trials by publication status and disease category.

Among the 26 analyzed RCTs, 6 trials were published, while 20 trials remained unpublished (Table 2). A comparison of trial characteristics between the published and unpublished groups revealed several significant differences in distribution. The data on the start time of the registration study showed a statistically significant disparity (*P* = 0.002). Notably, all published trials (9/9, 100%) were registered before 2009, whereas among unpublished trials, a majority (11/17, 64.71%) began in 2010 or later. Significant differences were also observed in recruitment scope (*P* = 0.032) and the number of centers (*P* = 0.038). Published trials were more likely to be international and multicenter compared to their unpublished counterparts. A notable difference in sample size distribution was present (*P* = 0.010), with 55.56% of published trials enrolling more than 100 participants versus 5.88% of unpublished trials. Moreover, within Neurological Disorders, epilepsy and Parkinson’s Disease were the most frequently evaluated targets, while obsessive-compulsive disorder predominated among trials of Psychiatric/Psychological Disorders; depression exhibited the highest publication rate across all conditions, whereas obsessive-compulsive disorder had the lowest publication rates with no corresponding published RCTs (Figure 2).

**TABLE 2:** Characteristics of RCTs related to DBS via publication status.

Characteristic	Published (9)		Not published (17)		*P*
**Start time**					0.002
Before 2009 (inclusive)	9	100.00%	6	35.29%	
After 2010 (inclusive)	0	0.00%	11	64.71%	
Condition					0.357
Neurological disorders	8	88.89%	11	64.71%	
Psychiatric/psychological disorders	1	11.11%	6	35.29%	
Study design					>0.999
Parallel group	7	77.78%	12	70.59%	
Non-parallel group	2	22.22%	5	29.41%	
No. of arms					0.529
2	9	100.00%	15	88.24%	
≥ 3	0	0.00%	2	11.76%	
Blinding					0.630
None/open label	3	33.33%	3	17.65%	
Single/double/triple/quadruple	6	66.67%	13	76.47%	
Recruitment					0.032
National	6	66.67%	17	100.00%	
International	3	33.33%	0	0.00%	
No. of centers					0.038
Single center	2	22.22%	12	70.59%	
Multicenter	7	77.78%	5	29.41%	
No. of participants					0.010
< 100	4	44.44%	16	94.12%	
> = 100	5	55.56%	1	5.88%	
Region of PI					0.661
North America	3	33.33%	4	23.53%	
Non-North America	6	66.67%	13	76.47%	
Funding					0.032
None or departmental	2	22.22%	0	0.00%	
Industry or other external	7	77.78%	17	100.00%	

PI, principal investigator.

**FIGURE 3:**
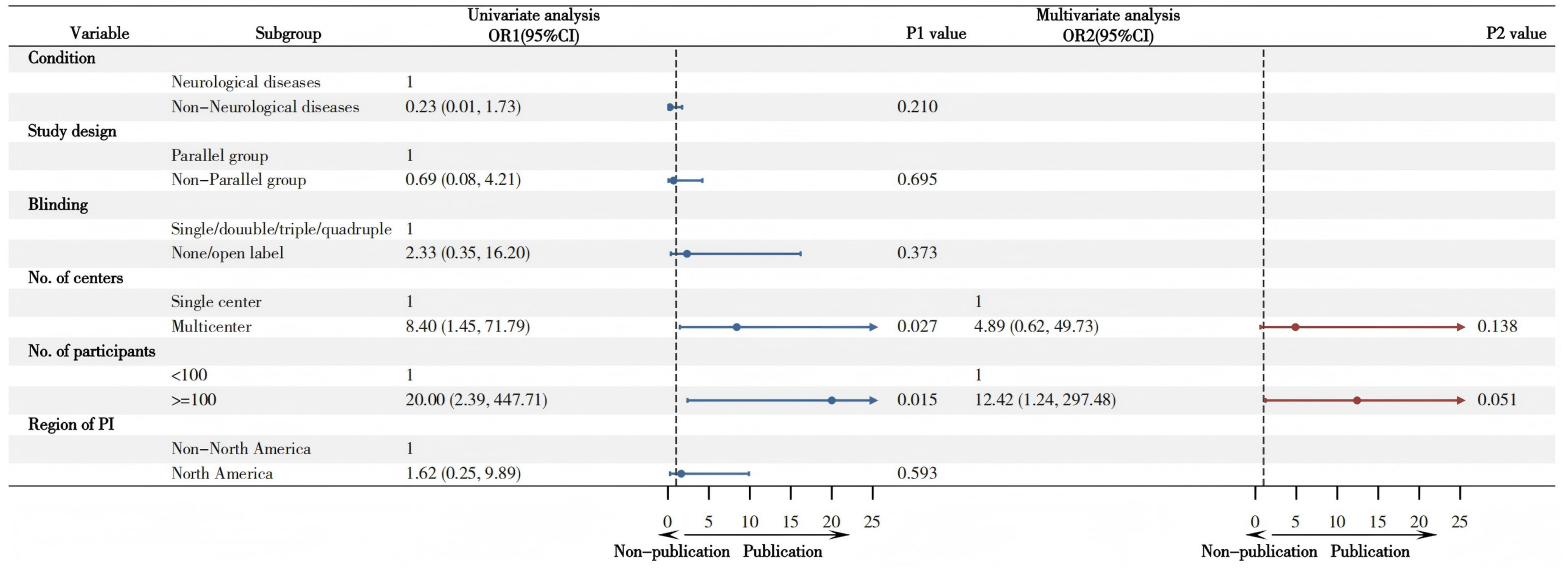
Forest plot of the effect of key study characteristics on publication status.

Logistic regression analysis was performed to identify factors associated with successful publication (outcome coded as 1; Figure 3). In univariate analysis, multicenter trials exhibited significantly higher odds of publication compared to single-center trials (OR = 8.40, 95% CI: 1.45–71.79, *P* = 0.027). Similarly, a larger sample size ( ≥ 100 participants) was strongly positively associated with publication (OR = 20.00, 95% CI: 2.39–447.71, *P* = 0.015). However, these associations did not remain statistically significant in the multivariate model. Furthermore, no other variables—including study design, blinding method, or the principal investigator’s region—were significantly associated with publication status.

### Reporting adequacy of published RCTs

Among the 9 published trials, 6 (66.67%) demonstrated adequate reporting according to the CONSORT 2010 checklist, while 3 (33.33%) were classified as inadequate (Supplementary Table S1). The adequately reported trials were characterized by several common, albeit non-significant, features: all were registered before 2010, a higher proportion focused on neurological disorders, used a two-arm design, and involved international recruitment, compared with the inadequately reported group. Both groups were predominantly multicenter studies with sample sizes exceeding 30 participants.

### Design flaws of published RCTs

**FIGURE 4:**
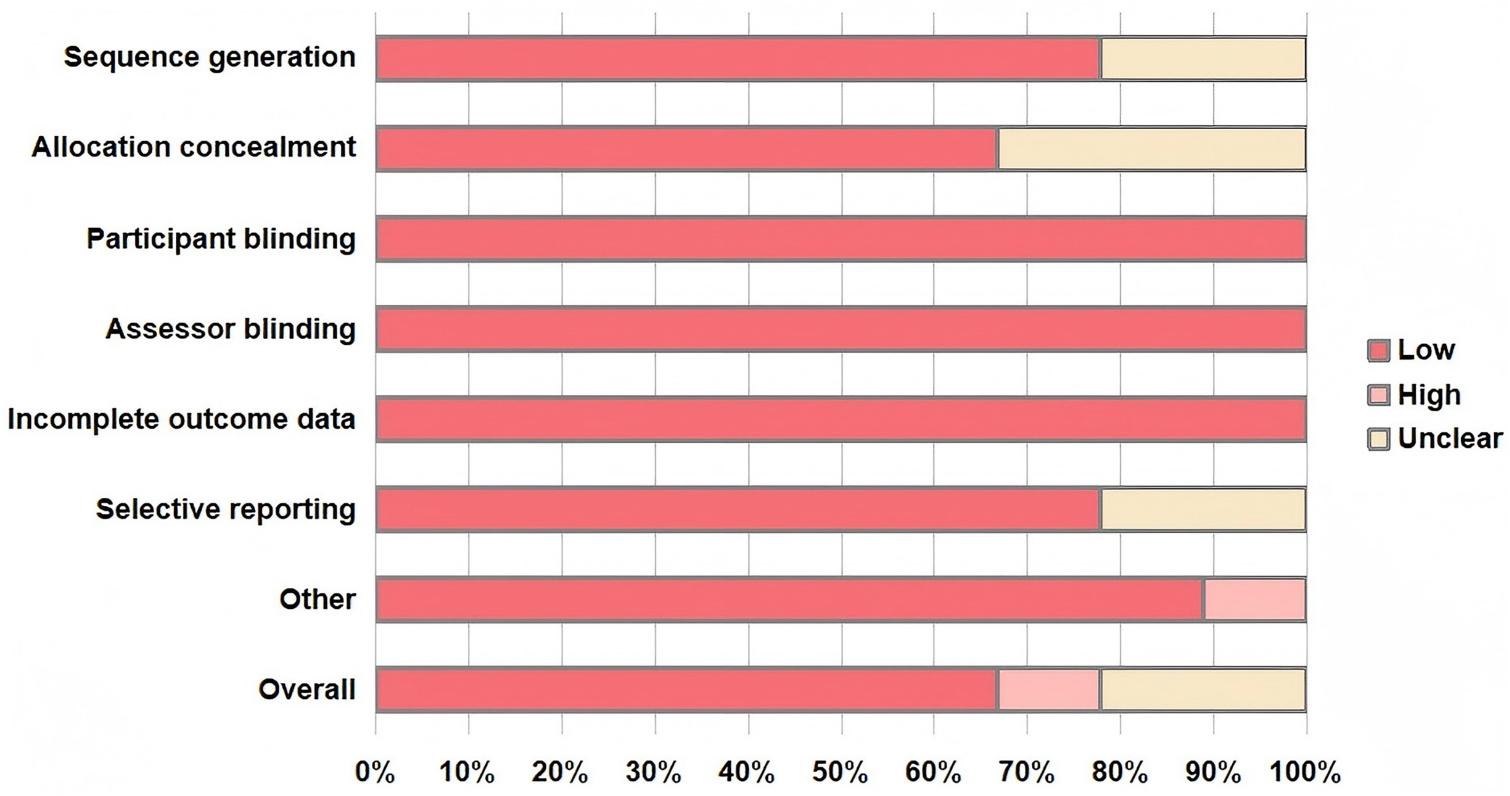
Risk-of-bias assessment.

The risk-of-bias assessment indicated generally high methodological quality, with key domains such as blinding and handling of outcome data showing 100% low risk (Figure 4). However, reporting deficiencies led to notable proportions of “unclear” judgments for randomization and selective reporting. Among the 9 published trials assessed for avoidable design flaws, the distribution of characteristics between trials with and without such flaws is presented in Supplementary Table S2. Due to the small sample size, no statistically significant differences were observed in any of the variables examined. All trials in both groups were registered before 2010. A higher proportion of trials without design flaws (6/6, 100%) focused on neurological disorders than those with flaws (2/3, 66.67%). The groups showed similar distributions in study design, blinding, number of centers, sample size, and investigator region.

### Research waste of RCTs

Among the 26 analyzed trials, 5 were free of research waste, while 21 exhibited at least one form of waste (Table 3). The distribution of trial characteristics between these two groups revealed several significant differences. All trials without waste (5/5, 100%) were registered before 2009, compared with fewer than half (11/21, 52.38%) of trials with research waste. The scope of recruitment was a particularly strong factor: all waste trials were nationally recruited, whereas 60% (3/5) of waste-free trials were international (*P* = 0.004). Furthermore, a higher proportion of non-waste trials were multicenter studies (80.00% vs. 38.10%), although this difference was not statistically significant (*P* = 0.148). No other variables, including the start time, target condition, study design, blinding, sample size, the principal investigator’s region, or funding source, were statistically significantly associated with research waste.

**TABLE 3:** Characteristics of published RCTs related to DBS via research waste.

Characteristic	Without research waste (5)		With research waste (24)		*P*
Start time					0.053
Before 2009 (inclusive)	5	100.00%	10	47.62%	
After 2010 (inclusive)	0	0.00%	11	52.38%	
Condition					0.278
Neurological disorders	5	100.00%	14	66.67%	
Psychiatric/psychological disorders	0	0.00%	7	33.33%	
Study design					>0.999
Parallel group	4	80.00%	15	71.43%	
Non-parallel group	1	20.00%	6	28.57%	
No. of arms					>0.999
2	5	100.00%	19	90.48%	
≥ 3	0	0.00%	2	9.52%	
Blinding					0.558
None/open label	2	40.00%	4	19.05%	
Single/double/triple/quadruple	3	60.00%	17	80.95%	
Recruitment					0.004
National	2	40.00%	21	100.00%	
International	3	60.00%	0	0.00%	
No. of centers					0.148
Single center	1	20.00%	13	61.90%	
Multicenter	4	80.00%	8	38.10%	
No. of participants					0.558
< 100	3	60.00%	17	80.95%	
≥100	2	40.00%	4	19.05%	
Region of PI					>0.999
North America	1	20.00%	6	28.57%	
Non-North America	4	80.00%	15	71.43%	
Funding					0.354
None or departmental	1	20.00%	1	4.76%	
Industry or other external	4	80.00%	20	95.24%	

PI, principal investigator.

**FIGURE 5:**
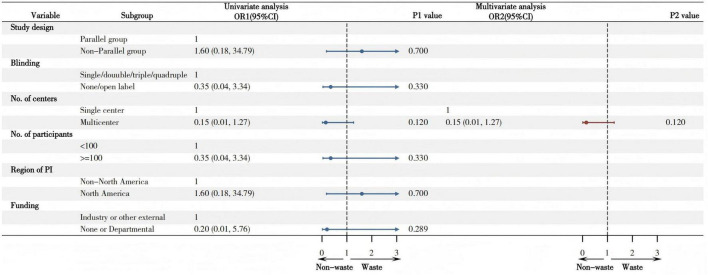
Forest map of the effect of key study characteristics on the presence of research waste.

Logistic regression analysis was conducted to identify factors associated with research waste (binary outcome; Figure 5). Univariable analysis revealed no significant associations between study characteristics and the occurrence of waste. Owing to the limited number of events, multivariable analysis was restricted to a single variable (number of centers). In this model, multicenter studies showed a nonsignificant trend toward lower odds of waste compared with single-center studies (unadjusted and adjusted ORs = 0.15; 95% CI, 0.01–1.27; *P* = 0.12).

## Discussion

This cross-sectional analysis provides a comprehensive overview of the characteristics and the extent of research waste in phase III and IV RCTs investigating DBS. Based on the included trials, our findings indicate that over half were initiated in or before 2009. The evidence base is predominantly focused on neurological disorders and characterized by a high degree of industry sponsorship. However, this research effort is significantly undermined by pervasive and high levels of research inefficiency, manifested primarily through non-publication and, to a lesser extent, through inadequate reporting and methodological shortcomings in the published literature.

The most striking finding is the exceptionally high non-publication rate of 65.38% among completed DBS RCTs. This magnitude of result attrition represents a severe loss of research investment and participant contribution. Notably, all successfully published trials were initiated in or before 2009. This pattern likely represents a period effect, wherein trials conducted during a peak period of efficacy confirmation for disorders encountered fewer barriers to completion. Conversely, the higher non-publication rate among more recently registered trials may reflect the growing complexity of exploring novel indications or paradigms, which often entails greater operational challenges and an increased likelihood of negative or inconclusive results (Davidson et al., 2024; Swinford-Jackson and Pierce, 2024; von Elm et al., 2008). The stark contrast between internationally collaborative, multicenter trials and nationally conducted, single-center studies underscores the critical role of research infrastructure in ensuring dissemination (Shen et al., 2024; Zhao et al., 2024).

Consistent with existing literature, the univariate analysis confirmed that larger, multicenter trials are significantly more likely to be published, likely reflecting greater resource availability, institutional pressure, and the scientific impact associated with collaborative efforts (Fei et al., 2026). However, the loss of statistical significance for these factors in the multivariate model is noteworthy. This attenuation suggests that the apparent advantage of multicenter designs and larger sample sizes may not be an independent driver of publication, but rather a proxy for other confounding factors or simply a reflection of the limited statistical power inherent in our small sample size (Li et al., 2026). Consequently, while resource-intensive trials appear to fare better, no single demographic or structural characteristic emerged as an independent predictor of successful dissemination, highlighting the complex and often non-transparent nature of publication bias in this field.

Among the published trials, the methodological quality, as assessed using the risk-of-bias tool, was generally robust, with key domains such as blinding and handling of incomplete outcome data consistently rated as “low risk” across all trials. However, the persistence of “unclear” risk judgments, especially regarding sequence generation and allocation concealment, points to a persistent deficit in transparent reporting. This deficiency prevents readers and systematic reviewers from fully appraising the internal validity of these trials. Furthermore, the fact that one-third of published trials were deemed to have inadequate reporting under CONSORT standards highlights that publication alone does not produce usable, high-quality evidence.

The high prevalence of research waste in DBS RCTs indicates a systemic inefficiency. Specifically, trials initiated later were more likely to involve research waste and were predominantly conducted in single-country settings rather than in international collaborations. This pattern further suggests that earlier research targeting established indications might face fewer hurdles, while more recent explorations of novel applications encounter greater challenges. Moreover, the complexity is exacerbated by the specific operational demands of neuromodulation research. Unlike pharmacological RCTs, DBS trials often require sham incisions or anesthesia, raising unique ethical dilemmas regarding equipoise and patient safety that frequently result in protocol amendments, recruitment failures, or excessive dropout rates, thereby contributing to research waste (Hinwood et al., 2022; Horng and Miller, 2015). Although well-designed sham-controlled trials are essential for establishing the efficacy of DBS and remain a methodological gold standard when clinical equipoise exists, the use of invasive sham procedures—which expose control participants to surgical risks while withholding potentially beneficial neurostimulation—has prompted intense ethical debate regarding the justification of sham surgery controls, particularly when effective alternatives exist (Horng and Miller, 2003; Wartolowska et al., 2014). In parallel, regulatory authorities and institutional review boards have introduced increasingly stringent requirements for sham surgery trials, mandating independent data safety monitoring boards, enhanced informed consent, and stricter risk–benefit justification (Hetzler et al., 2023; Tambone et al., 2017). These evolving regulatory and ethical expectations further compound the operational difficulty of conducting DBS RCTs.

In addition, the model assessing the composite outcome of research waste, which encompasses non-publication, inadequate reporting, and methodological flaws, showed no statistically significant independent associations with the variables examined. This indicates that the factors contributing to comprehensive research waste in this field are likely multifactorial and complex, and are not adequately captured by the common design and logistical characteristics included in our analysis. The lack of identifiable associations highlights the need to explore a wider range of potential contributing factors to fully understand the mechanisms underlying inefficiency in neuromodulation research.

It is noteworthy that the number of Phase III/IV DBS RCTs available for analysis was relatively small compared to the broader landscape of registered neurosurgical trials. This scarcity is not merely a methodological artifact but underscores a critical vulnerability in the evidence base for neuromodulation. While Phase I/II trials constitute the bulk of DBS research, the transition to late-phase RCTs may be fraught with operational and financial barriers (Mishra et al., 2024). Consequently, our findings specifically highlight that the waste occurring within this limited pool of advanced trials is particularly egregious, representing a failure at the very stage where clinical translation is supposed to be secured.

There are several limitations in our study. First, the total of 26 included RCTs, while representing a comprehensive set for the defined search criteria, constituted a limited sample size (Shieh, 2013). This constraint reduced the statistical power of subgroup analyses and logistic regression models, especially those performed exclusively on the subset of published trials. Second, the data source was confined to the ClinicalTrials.gov registry (Dai et al., 2025). Although it is the most extensive public trial registry, our reliance on it means that trials registered only in other databases or those never registered may have been omitted, which could influence the representativeness of our findings (Hopewell et al., 2007). Third, the operational definitions used for assessing reporting adequacy and design flaws were necessarily dichotomous. This approach facilitated analysis but may have oversimplified more subtle gradations in methodological quality. Finally, the observational and cross-sectional design of this analysis precludes causal inference. The associations identified between trial characteristics and outcomes, such as publication status or research waste, should be interpreted as correlational rather than causal (Lin et al., 2025).

## Conclusion

This study reveals a critical deficit in the translational pipeline of DBS research, characterized by an alarmingly high non-publication rate and significant research waste among completed phase III/IV RCTs. Our findings underscore that successful publication may be strongly associated with the historical context of the trial. The absence of independent predictors for research waste in the multivariate models suggests that inefficiency in DBS trials is driven by multifactorial complexities rather than isolated demographic or design characteristics, likely compounded by limited statistical power. Addressing this systemic inefficiency requires a paradigm shift toward mandatory results reporting, stricter enforcement of CONSORT guidelines, and increased investment in collaborative networks to ensure that the substantial investments in DBS research yield usable evidence for clinical practice.

## Data Availability

The original contributions presented in the study are included in the article/Supplementary material, further inquiries can be directed to the corresponding authors.
