# Supplementary material for: Research waste of randomized controlled trials related to deep brain stimulation: a cross-sectional analysis
Source: Front Aging Neurosci. 2026 Jul 10;18:1818434. doi: 10.3389/fnagi.2026.1818434 (PMC13395928; doi:10.3389/fnagi.2026.1818434)
Supplement: Supplementary file 1 [file Data_Sheet_1.pdf]

Table S1 Characteristics of published RCTs related to DBS via the CONSORT 2010 checklist

Characteristic	Adequate reporting (6)		Inadequate reporting (3)		P
**Start time**					>0.999
before 2009 (inclusive)	6	100.00%	3	100.00%	
after 2010 (inclusive)	0	0.00%	0	0.00%	
**Condition**					0.375
Neurological disorders	5	83.33%	2	66.67%	
Psychiatric/Psychological disorders	0	0.00%	1	33.33%	
**Study design**					>0.999
Parallel group	5	83.33%	2	66.67%	
Non-Parallel group	1	16.67%	1	33.33%	
**No. of arms**					0.083
2	6	100.00%	1	33.33%	
≥3	0	0.00%	2	66.67%	
**Blinding**					0.464
None/open label	3	50.00%	0	0.00%	
Single/double/triple/quadruple	3	50.00%	3	100.00%	
**Recruitment**					0.200
National	3	50.00%	4	133.33%	
International	3	50.00%	0	0.00%	
**No. of centers**					>0.999
Single center	1	16.67%	1	33.33%	
Multicenter	5	83.33%	3	100.00%	
**No. of participants**					>0.999
<100	3	50.00%	1	33.33%	
>=100	3	50.00%	2	66.67%	
**Region of PI**					0.226
North America	1	16.67%	2	66.67%	
Non-North America	5	83.33%	1	33.33%	
**Funding**					>0.999
None or Departmental	3	50.00%	1	33.33%	
Industry or other external	6	100.00%	2	66.67%	

PI: principal investigator.

Table S2 Characteristics of published RCTs Related to DBS via risk-of-bias Assessment

Characteristic	Absence design flaws (6)		Presence design flaws (3)		P
**Start time**					>0.999
before 2009 (inclusive)	6	100.00%	3	100.00%	
after 2010 (inclusive)	0	0.00%	0	0.00%	
**Condition**					0.333
Neurological disorders	6	100.00%	2	66.67%	
Psychiatric/Psychological disorders	0	0.00%	1	33.33%	
**Study design**					>0.999
Parallel group	5	83.33%	2	66.67%	
Non-Parallel group	1	16.67%	1	33.33%	
**No. of arms**					>0.999
2	5	83.33%	2	66.67%	
≥3	0	0.00%	0	0.00%	
**Blinding**					>0.999
None/open label	2	33.33%	1	33.33%	
Single/double/triple/quadruple	4	66.67%	2	66.67%	
**Recruitment**					0.464
National	3	50.00%	3	100.00%	
International	3	50.00%	0	0.00%	
**No. of centers**					>0.999
Single center	1	16.67%	1	33.33%	
Multicenter	5	83.33%	2	66.67%	
**No. of participants**					>0.999
<100	3	16.67%	1	33.33%	
>=100	3	83.33%	2	66.67%	
**Region of PI**					>0.999
North America	2	33.33%	1	33.33%	
Non-North America	4	66.67%	2	66.67%	
**Funding**					0.500
None or Departmental	2	33.33%	0	0.00%	
Industry or other external	4	66.67%	3	100.00%	

PI: principal investigator.
